# Solitary Cervical Plasmacytoma Presenting as Acute Quadriparesis Following a Recent Cerebral Infarct: A Case Report

**DOI:** 10.7759/cureus.93462

**Published:** 2025-09-29

**Authors:** Venkat Nag Pratap Reddy Avula, Sowmya Gopalan, Preetam Arthur

**Affiliations:** 1 General Medicine, Sri Ramachandra Institute of Higher Education and Research, Chennai, IND

**Keywords:** cerebrovascular accident, cervical spine, quadriparesis, solitary plasmacytoma, spinal cord compression

## Abstract

Solitary plasmacytoma of bone (SPB) is a rare plasma-cell neoplasm that can cause acute neurological compromise, especially when involving the cervical spine. We report a 59-year-old male with a recent right hemispheric hemorrhagic infarct who developed rapidly progressive quadriparesis and urinary retention 15 days later. Neurological examination showed spastic quadriparesis with a C5 sensory level. MRI and CT revealed a pathological C4-C5 wedge compression fracture with cord compression. Emergency anterior C4-C5 corpectomy with fusion was performed. Intraoperative tissue culture grew *Staphylococcus aureus* and was treated with vancomycin. Histopathology confirmed a kappa-restricted plasma-cell neoplasm. Bone marrow biopsy showed less than 10% plasma cells, and PET-CT excluded systemic disease, confirming SPB. The patient subsequently received definitive radiotherapy (43.2 Gy in 24 fractions) and physiotherapy, resulting in partial motor recovery. This case highlights that new or worsening neurological deficits in post-stroke patients should prompt urgent spinal imaging to exclude compressive lesions. Early surgical decompression combined with radiotherapy and rehabilitation can preserve function and improve outcomes.

## Introduction

Solitary plasmacytoma of bone (SPB) is a rare, localized plasma-cell neoplasm, accounting for fewer than 5% of plasma-cell dyscrasias [1]. It most often involves the axial skeleton, with the thoracic spine being the most frequent site; cervical involvement is uncommon but carries a high risk of acute neurological compromise due to the confined spinal canal [2]. SPB is defined by a solitary bone lesion, histologic evidence of clonal plasma cells, and absence of systemic myeloma features (CRAB criteria - calcium elevation, renal failure, anemia, bone lesions) [3].

Diagnosis requires a high index of suspicion, especially when neurological symptoms evolve rapidly [4]. In this report, we describe an unusual presentation in which a cervical plasmacytoma was unmasked by quadriparesis occurring soon after a hemorrhagic stroke, emphasizing the need to reconsider new deficits in post-stroke patients.

## Case presentation

A 59-year-old male, a chronic smoker and alcohol-dependent, presented with sudden giddiness following a fall. Examination revealed mild gait imbalance without focal limb weakness. MRI brain demonstrated acute hemorrhagic infarcts in the right frontal, posterior temporal, occipito-parietal, and centrum semiovale. Cardiovascular workup, including echocardiography and carotid Doppler, was unremarkable. He had mild neck and shoulder pain, which was attributed to the cerebrovascular accident. He was managed conservatively with a single antiplatelet agent and statin and was discharged with mild residual unsteadiness.

Fifteen days later, he returned with rapidly progressive weakness of all four limbs and urinary retention. On examination, power was Medical Research Council (MRC) grade 3/5 in both the upper limbs and 2/5 in both the lower limbs, with hyperreflexia, spasticity, bilateral Babinski sign, and a C5 sensory level.

**Figure 1:**
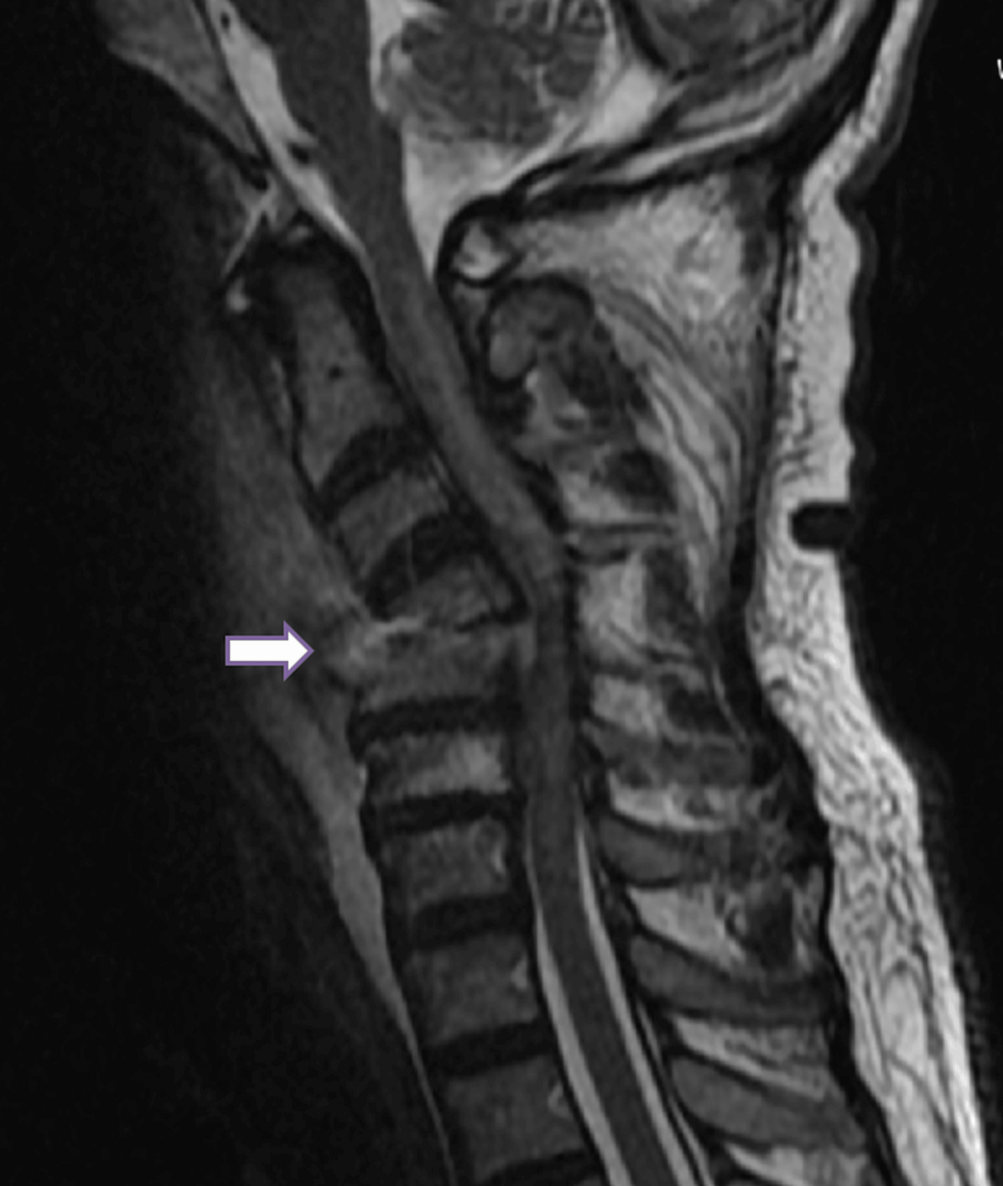
Sagittal T2-weighted MRI of the cervical spine. Collapse of the C5 vertebral body with heterogeneous marrow signal, posterior retropulsion, and severe spinal cord compression at the C4–C5 level (white arrow). Intramedullary T2 hyperintensity within the cord indicates edema.

MRI cervical spine revealed a pathological wedge compression fracture at C4-C5, prevertebral and paravertebral soft tissue masses, and severe spinal cord compression, and surrounding edema was seen in high T2 intramedullary signal. Figure 1 presents the sagittal T2-weighted MRI of the cervical spine.

CT confirmed bony destruction of C4 and C5 vertebral bodies. Given acute cord compression with instability, the patient underwent emergency anterior C4-C5 corpectomy and posterior cervical decompression and stabilization using standard pedicle screw and rod instrumentation with plate. Intraoperatively, infiltrating tumor tissue replacing vertebral bodies was noted. Intraoperative tissue cultures (multiple samples) grew *Staphylococcus aureus*. This was considered to represent a true superimposed infection rather than contamination, and intravenous vancomycin was administered, which was later changed to oral linezolid and given for a total duration of six weeks.

**Figure 2:**
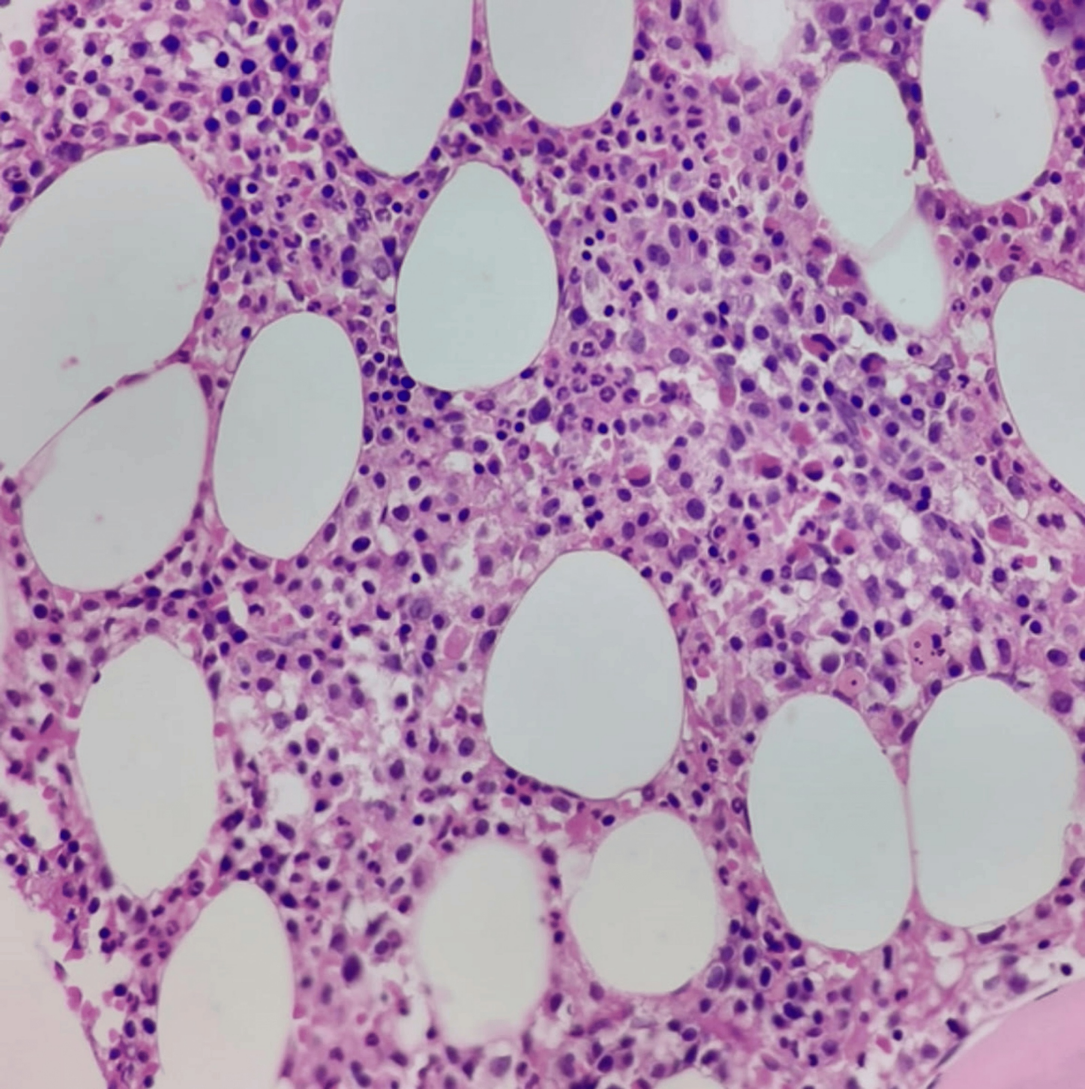
Bone marrow biopsy (H&E). High-power view (×400) showing diffuse plasma cell infiltration with eccentric nuclei, consistent with plasmacytoma.

Histopathology revealed sheets of atypical plasma cells with eccentric nuclei. Figure 2 presents the bone marrow biopsy (H&E).

Immunohistochemistry demonstrated CD138 positivity and kappa light-chain restriction. Systemic evaluation included a bone marrow biopsy (4% plasma cells), normal calcium, creatinine, and hemoglobin levels, and serum protein electrophoresis showing a small M-spike. PET-CT revealed no additional lesions, fulfilling diagnostic criteria for solitary plasmacytoma [3,5].

The patient received conformal radiotherapy (43.2 Gy in 24 fractions) to the cervical spine. Intensive physiotherapy and rehabilitation were initiated. At three-month follow-up, he had regained independent sitting balance and partial limb strength (bilateral upper limbs 4/5, bilateral lower limbs 3/5) [6]. He continues to receive physiotherapy for residual deficits. Our surveillance plan includes serial clinical examinations, laboratory monitoring (serum protein electrophoresis, immunofixation, and free light chain assay), and periodic whole-body imaging to detect any progression to multiple myeloma. At present, there is no evidence of disease recurrence or systemic progression.

## Discussion

SPB is a localized plasma-cell malignancy that can progress to multiple myeloma in up to 50-60% of cases over 10 years [1]. Cervical lesions are rare but may present with pain, instability, or acute myelopathy [2].

In this patient, a recent stroke masked early spinal symptoms, delaying detection until cord compression caused quadriparesis. The intraoperative isolation of *S. aureus *raised the possibility of contamination versus true infection. Given the consistent growth in multiple samples and clinical settings, we considered it a superimposed infection and treated the patient accordingly. Importantly, the radiological and histopathological findings remained diagnostic of plasmacytoma, and the infection did not alter the underlying diagnosis.

MRI is the imaging modality of choice, while PET-CT helps detect occult or systemic disease and assess treatment response [2,5]. Diagnostic criteria include histologic confirmation, normal marrow (<10% plasma cells), absence of myeloma-defining events, and no additional lesions on skeletal survey or PET-CT [3,5].

Management of SPB involves definitive radiotherapy (40-50 Gy in fractions) to achieve local control rates exceeding 80% [6]. Surgery is indicated for instability, pathological fractures, or progressive deficits, as in this case [4]. Post-treatment monitoring with serum protein electrophoresis, free light-chain assays, and periodic imaging is essential for early detection of progression [5].

Cervical plasmacytomas are rare, and cases mimicking cerebrovascular events are exceptionally uncommon. Prior reports have described cervical lesions presenting with stroke-like deficits, underscoring the importance of biopsy for accurate diagnosis [7]. Our case contributes to this limited literature by highlighting not only a diagnostic mimic of stroke but also the added complexity of concurrent infection.

This case illustrates that new or worsening neurological deficits in post-stroke patients should prompt evaluation for structural spinal pathology, even if symptoms appear temporally related to cerebrovascular events.

## Conclusions

In patients with recent stroke, rapidly evolving quadriparesis warrants urgent spinal imaging to exclude compressive lesions. Solitary cervical plasmacytoma, though rare, should be part of the differential diagnosis. Early surgical decompression combined with radiotherapy and rehabilitation can preserve function and improve quality of life.
